# Drivers and Cultural Influences on Healthcare‐Seeking Behaviors Among Ethnic Minority Older Adults in Rural Southwestern China: A Descriptive Qualitative Study

**DOI:** 10.1002/hsr2.71483

**Published:** 2025-11-09

**Authors:** Liutao Wu, Runxi Xiao, Yu Wu, Qingmei Huang, Yan Liang

**Affiliations:** ^1^ Fudan University School of Nursing Shanghai China

**Keywords:** ethnic minority, healthcare‐seeking behavior, older adults, qualitative research

## Abstract

**Background and Aims:**

Understanding the healthcare‐seeking behaviors of minority older adults is crucial for advancing health equity and enhancing health outcomes. To date, no qualitative research has specifically examined the healthcare‐seeking behaviors of ethnic minority older adults in China. The study seeks to elucidate the driving factors and cultural influences on health‐seeking behaviors among ethnic minority older adults in rural Southwestern China.

**Methods:**

Guided by the integrated symptom‐response framework—which explains how individuals interpret and respond to symptoms—a qualitative descriptive study was conducted, involving semi‐structured interviews with sixteen adults. The study's interview guide and thematic analysis were informed by this theoretical perspective.

**Results:**

Five themes emerged from the study. These included (1) self‐care for minor ailments, (2) seeking healthcare for serious conditions, (3) interpretation of health‐related factors, (4) understanding of diseases/symptoms within traditional ethnic cultural contexts, and (5) dynamic considerations based on experience and reality.

**Conclusion:**

The findings of this study offer critical insights for healthcare professionals and policymakers on the cultural determinants impacting the healthcare‐seeking behaviors of ethnic minority older adults. By understanding these factors, targeted interventions and policies can be implemented to enhance cultural sensitivity within healthcare services.

## 1 Introduction

Older adults from racial and ethnic minorities face persistent and pervasive health disparities. Older adults encounter several physical and mental health challenges and use healthcare services at higher rates than younger populations [1]. Meanwhile, older adults still have unmet needs and access to inadequate healthcare services [2]. Ethnic minorities, hindered by various structural, cultural, and socioeconomic barriers, are directly and indirectly experiencing deprived access to healthcare services [3].

Healthcare‐seeking behavior refers to any activity undertaken by individuals who believe they have a health problem or illness to seek appropriate treatment [4]. Understanding the healthcare‐seeking behavior of ethnic minority older adults is crucial for creating multisectoral changes for good health and well‐being and reducing inequalities. Previous studies have revealed several obstacles involving healthcare‐seeking for older members of ethnic minority patients, including language and illiteracy as critical barriers to accessing health information, communication behaviors, ethnic identity, and social norms [5]. However, we still know little about how cultural values and backgrounds may impact ethnic minority older adults' healthcare‐seeking behaviors in different ways, especially for minority older adults from rural areas.

Previous research showed that, for older people in rural China, cultural devaluation and structural constraints downgraded their expectation of receiving medical care, which was expressed as “unworthy of care and treatment” (“buzhide” in Chinese). Due to historical factors, disadvantaged ethnic minorities tend to be distributed in remote rural areas in China [6]. For example, the Dong nationality, one of the 55 ethnic minorities in China with a population of around 3 million, is distributed in the mountainous area in Southwestern China [6]. Older people in rural China almost reside in a less privileged environment with poorer educational and social support than their urban counterparts [7]. Older people's Healthcare‐seeking behaviors in rural areas occur in a complex social, institutional, and structural context [8]. In ethnic minority villages, unique ethnic and regional living habits and cultural customs have been formed by living conditions, production, and lifestyles [9]. Therefore, there is a need to understand the drivers for and cultural influences on healthcare‐seeking behaviors among ethnic minority older adults in rural China to develop tailored interventions.

Healthcare‐seeking behavior has evolved to understand how people use the healthcare systems in their socio‐cultural, economic, and demographic circumstances [10]. Previous studies have explored the healthcare‐seeking behaviors of older adults in rural areas from a singular perspective, such as healthcare utilization or healthcare provider choice [11]. Additionally, these studies have primarily focused on exploring the relationship between healthcare‐seeking behaviors and factors such as background, socioeconomic status, and health status [11, 12]. However, a comprehensive understanding of the healthcare‐seeking behaviors of older adults residing in traditional minority villages remains limited. Addressing this gap is crucial, as ethnic minority older adults in rural China often experience compounded healthcare disparities due to structural inequities, cultural marginalization, and limited access to culturally competent services—factors that collectively undermine health outcomes and equity. Therefore, there is a need to understand the drivers for and cultural influences on healthcare‐seeking behaviors among ethnic minority older adults in rural China to develop tailored interventions.

The integrated symptom‐response framework proposed by Wyke et al. [13] provides a comprehensive guide for us to deeply understand how individuals explain, evaluate, and act when symptoms or diseases occur. This framework integrates the disease action model, the commonsense model of health and disease self‐regulation, and the network event model, providing an integrative perspective for an in‐depth understanding of the emotional context, cognitive response, and interaction with social networks in healthcare‐seeking behavior. Guided by the integrated symptom‐response framework [13], this study will conduct in‐depth interviews with older adults in traditional minority villages to describe how they act, interpret, and evaluate their healthcare‐seeking behaviors, as well as how individuals interact with their cultural and social structures. This study will contribute new knowledge to a comprehensive understanding of the healthcare‐seeking behaviors of minority older adults and provide theoretical perspectives and practical implications for promoting the health of minority older adults.

## 2 Methods

### 2.1 Study Design

In this study, a descriptive qualitative design was utilized due to its capacity to delve into nursing‐related phenomena, elucidate the essence of experiences, and glean insights from primary informants on a less understood phenomenon [14]. Specifically, semi‐structured and in‐depth interviews were employed to probe into the healthcare‐seeking behaviors of ethnic minority older adults in rural southwestern China. Thematic analysis was employed in this descriptive qualitative study to identify patterns in the interview data, systematically coding and analyzing themes related to the drivers and cultural influences affecting healthcare‐seeking behaviors. The qualitative study's reporting adhered to the COREQ (Consolidated Criteria for Reporting Qualitative Research) checklist, guaranteeing thorough and transparent documentation of the research process.

### 2.2 Setting

This study was conducted in the traditional Dong ethnic villages of Rongjiang and Jinping Counties, situated in Qiandongnan Prefecture, Guizhou Province, China. These regions are predominantly inhabited by the Dong ethnic group, one of China's 55 recognized ethnic minorities, with a population of around 3 million [15]. The Dong community primarily resides in the mountainous areas of Southwestern China, where rural settings present significant challenges in accessing healthcare. Older adults belonging to ethnic minorities in these areas encounter multiple barriers, including cultural practices, structural constraints, and economic limitations, which influence their healthcare‐seeking behaviors [16]. The distinct cultural customs and lifestyle habits of the Dong people, shaped by their environment, further influence their healthcare approaches.

### 2.3 Participants and Recruitment

The recruitment process took place over a period of 3 months with participants being recruited from traditional Dong ethnic villages in Rongjiang County and Jinping County, located in Qiandongnan Prefecture, Guizhou Province, China, the largest region inhabited by the Dong ethnic group in China. A purposeful sampling strategy was adopted, utilizing both stratified and maximum variation sampling to ensure broad representation and capture diverse participant experiences.

Researchers provided in‐person explanations of the study's objectives and methodologies to potential participants, after which written informed consent was obtained from those who agreed to take part. To ensure understanding among participants with limited literacy, the consent process was conducted verbally in the local Dong language, using simplified language and examples. Trained researchers read the consent form aloud, answered questions, and confirmed comprehension before participants signed the form. Once consent was secured, participants were enrolled, and interviews were conducted.

To guarantee diversity and heterogeneity, the recruitment process specifically targeted older adults from varying socio‐demographic backgrounds, with attention to factors such as age, gender, education level, and living situation. The inclusion criteria comprised (a) individuals aged 60 years and above, (b) who were residents of Dong nationality villages and (c) demonstrated normal communication abilities. Exclusion criteria encompassed individuals unable to complete the interview due to physical limitations. Recruitment continued until data saturation was reached, indicated by the absence of new themes or insights in further interviews. A total of 16 participants were interviewed, including seven males and nine females, with an average age of 73.2 years.

### 2.4 Ethical Approval and Consent

Ethical approval for this study was obtained from the Ethics Committee of the School of Nursing, Fudan University (IRB Number: TYSQ 2021‐03‐03), ensuring adherence to ethical standards and the principles set forth in the Declaration of Helsinki (World Medical Association, 2013). Before commencing interviews, we ensured that both oral and written informed consent were obtained. To protect the confidentiality of participants, no personal details were recorded in any of our records, such as interview transcripts. Findings were shared anonymously, and access to data was limited to the research team exclusively.

### 2.5 Data Collection

Data collection took place from July to September 2021. Utilizing a broad inquiry, investigators prompted participants to describe their experiences: “Can you share the details of the last time you experienced discomfort?” Subsequent interview questions focused on elucidating the motivators and cultural impacts on their healthcare‐seeking behaviors. A semi‐structured interview guide was formulated by the research team after reviewing the literature and conducting preliminary interviews. Provided below are samples of interview outlines: “When you are unwell, how do you typically handle your discomfort? What specific methods do you utilize to improve your condition, and what outcomes have you experienced after employing these strategies? What made you decide on these methods to relieve your discomfort? When looking for these methods, which factors played the most important role in your decision‐making process? Can you explain why these factors had such a strong impact? What thoughts and feelings did you have while attempting to find methods to relieve the discomfort?” At the same time, the interview techniques of probing, guiding, and encouraging were used to further explain each question and tell the details and feelings in the process of health‐seeking behaviors. Demographic data were collected before the interviews. 16 face‐to‐face interviews were conducted and audio recorded by trained researchers at a private location selected by the participants, predominantly in their homes or a designated village room, ensuring a secure, hospitable, and conducive atmosphere for information sharing.

The audio recordings of the interviews, lasting between 20 and 50 min each, were transcribed into a Word document by investigators fluent in the Dong language due to the unavailability of transcription tools. The first and third female authors, who both had a background related to the Dong ethnic group and experience in qualitative study, cross‐checked the transcriptions by replaying the recordings. The second author, who had a Ph.D. in nursing and extensive experience in qualitative research, served as consultant for data analysis. The demographic information of the interviewees was then inputted into a standardized datasheet and managed using Excel 2003.

### 2.6 Data Analysis

Thematic analysis was conducted following Braun and Clarke's (2012) guidelines and guided by the Integrated Symptom‐Response Framework [13], which integrates the Illness Action Model, the Common Sense Model of Self‐Regulation, and the Network Episode Model to elucidate individuals' responses to symptoms. This framework underscores the significance of symptom recognition, interpretation, and response processes influenced by knowledge, social interactions, emotions, as well as cultural and structural factors.

An a priori codebook was established with predefined codes and definitions. Emergent codes, identified through meticulous examination of transcripts and field notes, were integrated into the existing codebook.

The research team systematically categorized and merged conceptually linked codes, achieving consensus on the final themes. These themes were structured, defined, and harmonized with the theoretical framework, substantiated by quotations from participants. Subsequent interpretations were formulated, focusing on how healthcare‐seeking behaviors of ethnic minority older adults were molded by their actions, interpretations, and assessments.

To ensure qualitative rigor, methodologies consistent with Guba's [17] principles of trustworthiness were implemented. Regular team meetings were conducted to assess code applications, and individuals not involved in coding were enlisted to scrutinize and question coding decisions, thereby furnishing external validation of the analytical process.3.Findings.

### 2.7 Participant Demographics

A total of 16 participants, comprising 9 females and 7 males aged between 64 and 83 years, were interviewed. Among the participants, the majority had education levels below primary school (*n* = 9), with fewer having completed primary school (*n* = 6) and only one possessed a junior college degree. In terms of living arrangements, a significant portion resided with their spouse and/or children (*n* = 10), while others lived alone (*n* = 2) or with solely their children (*n* = 4). While most participants reported being in general or good health (*n* = 15), a high prevalence of chronic diseases was observed, with the majority having hypertension (*n* = 10) and others reporting conditions such as arthritis, rheumatic diseases, and chronic obstructive pulmonary disease. It is noted that all participants were covered by medical insurance. Table 1 shows the demographic characteristics.

**Table 1:** Demographic characteristics of the sample (*n* = 16).

Number	Gender	Age	Marital status	Education background	Residence status	Health status	Medical insurance	Any chronic diseases
P1	Female	64	Married	Below primary school	Live with spouse and children	Good	Yes	Yes, hypertension
P2	Male	70	Married	Primary school	Live with spouse and children	General	Yes	Yes, hypertension
P3	Female	70	Widowed	Below primary school	Live only with children	Good	Yes	Yes, hypertension
P4	Female	79	Widowed	Below primary school	Live alone	General	Yes	Yes, hypertension
P5	Male	73	Married	Primary school	Live only with children	Good	Yes	No
P6	Male	68	Married	Primary school	Live with spouse and children	Good	Yes	No
P7	Female	74	Widowed	Primary school	Live only with children	General	Yes	No
P8	Female	69	Married	Below primary school	Live only with children	General	Yes	Yes, hypertension
P9	Female	81	Married	Below primary school	Live with spouse and children	General	Yes	Yes, arthritis, rheumatic diseases
P10	Male	83	Married	Junior college	Live with spouse only	General	Yes	Yes, chronic obstructive pulmonary disease
P11	Female	75	Married	Below primary school	Live with spouse only	General	Yes	Yes, hypertension
P12	Male	66	Married	Primary school	Live with spouse only	General	Yes	Not clear
P13	Female	69	Married	Primary school	Live with spouse only	General	Yes	Not clear
P14	Male	81	Married	Below primary school	Live with spouse and children	General	Yes	Not clear
P15	Male	75	Married	Below primary school	Live alone	General	Yes	No
P16	Female	74	Widowed	Below primary school	Live only with children	General	Yes	Yes, hypertension

### 2.8 Thematic Analysis

**Figure 1:**
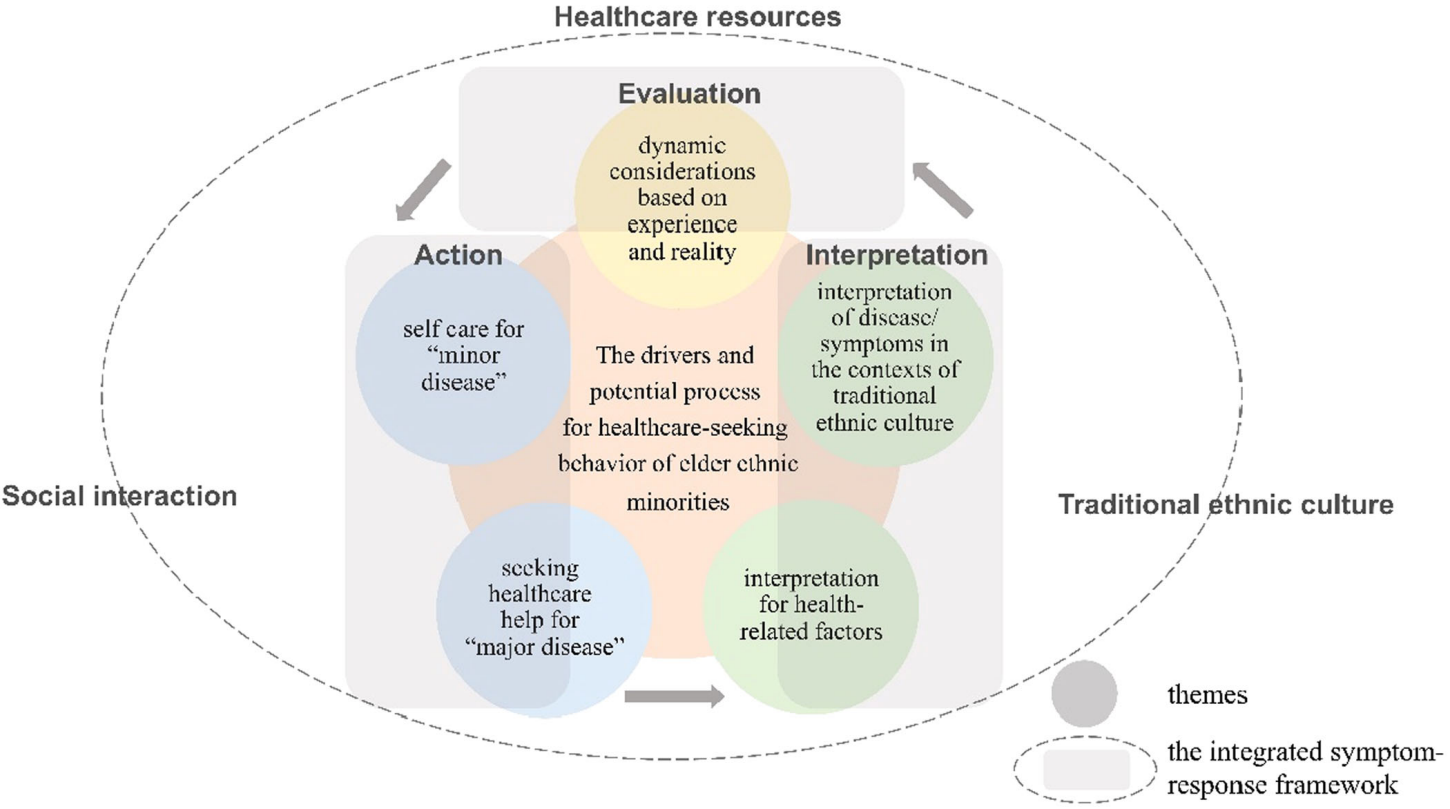
The drivers and potential process for healthcare‐seeking behaviors of a population of older Dong Chinese: based on the integrated symptom‐response framework.

**Figure 2:**
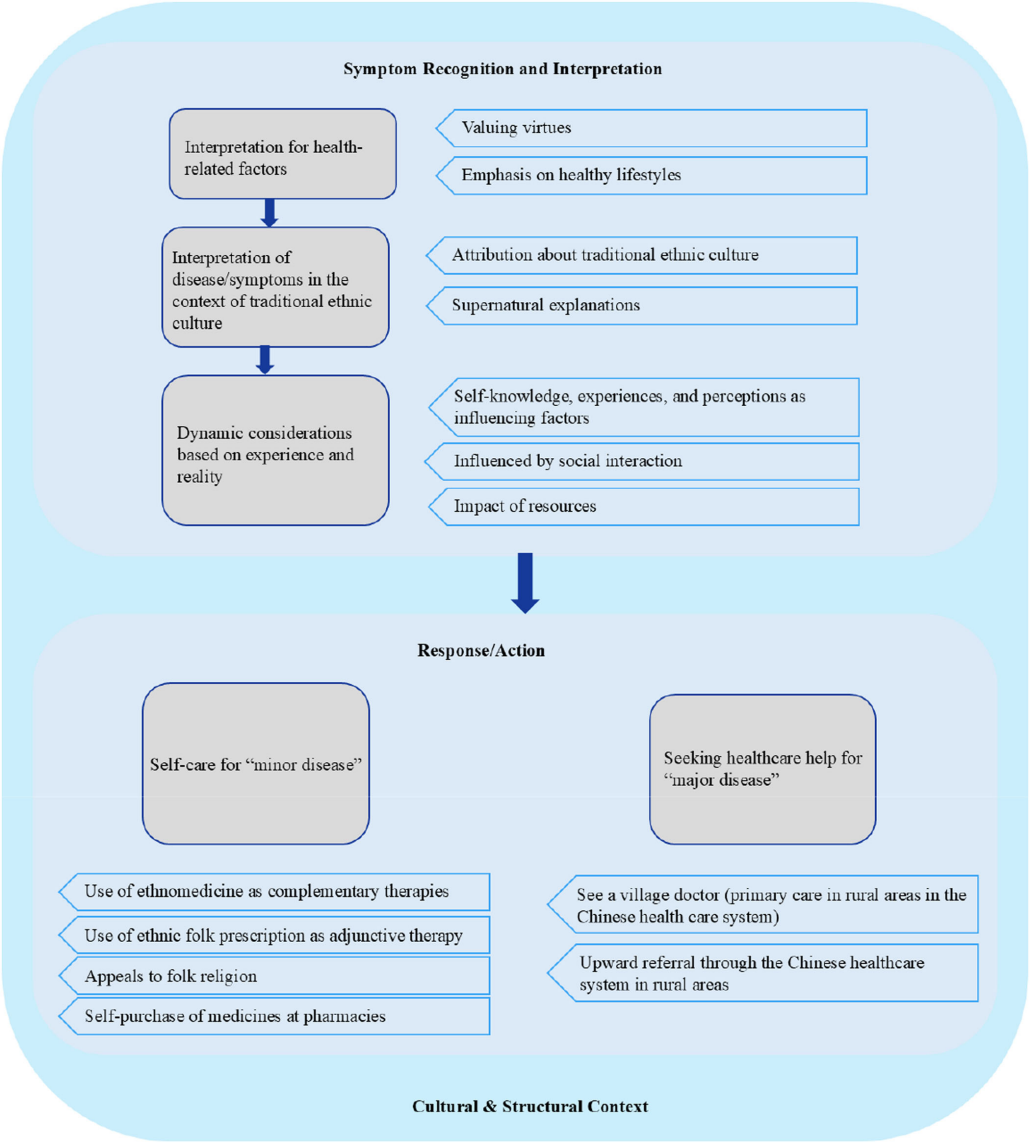
Overview of categories and themes.

The themes extracted during the analysis are outlined in Figure 1. Five key themes were extracted as influencing healthcare‐seeking behaviors among ethnic minority older adults: (1) self‐care for minor ailments, (2) seeking healthcare for serious conditions, (3) interpretation of health‐related factors, (4) understanding of diseases/symptoms within traditional ethnic cultural contexts, and (5) dynamic considerations based on experience and reality. Additionally, the study delineates the potential process through which actions, interpretations, and evaluations of ethnic minority older adults influence their healthcare‐seeking behaviors, as illustrated in Figure 2.

#### 2.8.1 Theme 1: Self‐Care for Minor Ailments

The participants expressed a preference for self‐care in managing minor ailments, incorporating traditional ethnomedicine as complementary therapies, ethnic folk remedies as supportive treatments, seeking solace in folk religion, and purchasing medicines directly from pharmacies. These actions form part of a comprehensive problem‐solving approach, with the decision to seek care contingent upon symptom severity and influenced by ingrained traditional cultural practices.

At the onset of feeling unwell, the participants indicated a tendency to prioritize local ethnomedical remedies like traditional Chinese medicine, medicinal wine, and scrape therapy (gua sha).

Feeling sick? No worries, just go pick some herbs and make yourself a nice stew.

(P9)

We could whip up some medicated wine or something to help with the discomfort. And if you're starting to feel under the weather or have some aches, you should also give scraping a try.

(P13, P14)

They additionally employ traditional ethnic remedies, such as the utilization of medicine bags, silver jewelry, or copper coins, the act of “begging for food,” and the custom of throwing food outside the residence.

Make sure the kids wear medicine bags if they get sick a lot, and throw on some silver bracelets or copper jewelry for good measure.

(P1, P2)

When your body feels weak, eating food brought from afar might do you good.

(P4)

When you toss some rice outside, leave a dish or two along with a bowl of rice as a sign of respect to the ‘ghost.’ Then, call out the patient's name to ensure their spirit is safe from being taken away.

(P11)

Furthermore, during interviews, elders underscored the profound importance of fortune‐tellers and spirits in Dong culture. They explained that these entities play a crucial role in helping community members address and overcome ailments through spiritual interactions with elements of nature, including the veneration of stones, ancient trees, bridges, and wells. The elders emphasized the practices of “mending the good spirits” and “driving away the evil spirits” as integral aspects of their spiritual healing rituals.

So, apparently, if you pay your respects to an ancient tree, it can help keep you in good health. And if you're dealing with a pesky fever, giving some love to wells and bridges could help ease your symptoms.

(P9)

Whenever an old person is super weak, they'll request a fortune‐teller to swing by their place and ‘add some blessings.' I've been asked to do that a bunch of times now.

(P4)

I might consider inviting a fortune‐teller to come over and help dispel any evil spirits in my home by performing some rituals.

(P1,P12)

#### 2.8.2 Theme 2: Seeking Healthcare for Serious Conditions

The second prominent theme identified in the data pertained to the effort to obtain medical assistance for severe ailments. Participants proactively sought assistance from a village doctor when their physical symptoms deteriorated, demonstrating a willingness to engage with healthcare providers.

If we get really sick and can't manage it on our own, we'll see a doctor.

(P12)

After deciding to seek medical treatment, participants mentioned a tendency to follow the village‐town‐county referral sequence. This process involves interactions with a village doctor (representing primary care in rural areas of China's healthcare system) and progression through the hierarchical framework of the rural Chinese healthcare system. The choice of this course of action was shaped by the availability of healthcare resources and individuals’ grasp of symptom significance and potential outcomes.

I had a fever for the first time, and no matter what I tried, nothing seemed to work. I didn't want to risk waiting, so I went to see the village doctor. He gave me a prescription, but it didn't help, so he arranged a car to take me to the town hospital. I stayed there for a while, but my condition didn't improve, and the doctor recommended that I be transferred to the county hospital.

(P10)

#### 2.8.3 Theme 3: Interpretation of Health‐Related Factors

In this study, older individuals expressed a belief that health is shaped by cultural norms regarding well‐being. They placed importance on virtuous behavior and underscored the significance of maintaining a healthy lifestyle. Participants perceived virtues as a crucial element influencing one's health outcomes.

As individuals, we focus on ‘cultivating good causes (Xiuyin) to yield good results.’ By doing this, everything else falls into place. ‘Cultivating good causes(Xiuyin)’ means caring for the elderly and children, respecting seniors as we would our own parents, and showing kindness to the elderly, young, and those less fortunate. When you nurture these ‘good causes’ you'll naturally reap true ‘good results.’

(P1)

When it comes to health aspects, a significant number of older individuals emphasize the significance of maintaining proper nutrition, adequate sleep, regular exercise, and a positive mood. Moreover, they underscore the importance of the environment.

Eat well, drink well, live happily, and stay healthy…

(P9)

I believe fresh air, doing the right thing, eating well, and getting good sleep are important. People can't just sit around all the time; they need to stay active.

(P12)

#### 2.8.4 Theme 4: Understanding of Diseases/Symptoms Within Traditional Ethnic Cultural Contexts

Within the scope of this study, older adults construe diseases or symptoms through the lens of traditional Dong culture, associating them with traditional ethnic beliefs and supernatural rationales, potentially shaping the understanding of symptoms and their corresponding healthcare‐seeking actions.

The older Dong generation holds the belief that the act of “owing debts” and commemorating deceased relatives can result in feelings of unwellness.

I've heard from the older generation that if you owe someone a debt—whether it's money or food—and don't repay it in time, it can actually cause physical discomfort. The more you owe, the worse the symptoms get.

(P9)

Last night, I dreamt about my sister who passed away. Every time I do, it gives me such a bad headache that I can barely get out of bed.

(P1)

When family members who have passed miss you, it affects you and can make you feel physically ill.

(P15)

When older adults experience unexplained bodily symptoms or unfamiliar phenomena that lead to discomfort, they often attribute these occurrences to impurity.

Sudden sharp pains, long‐term illness, or unexpected weakness could be caused by a ‘ghost.’ Strange symptoms, like sudden discomfort or severe pain while traveling, might also be linked to ‘ghosts.’

(P9)

Going out and encountering strange or unclean things—basically, things that are like ghosts—can also lead to physical discomfort. For example, if snakes, frogs, or other creatures show up at your house, their presence might unsettle your spirit and make you feel uneasy.

(P15)

If you have dreams or nightmares that are hard to understand, it's best to consult a fortune teller to help you interpret them.

(P11)

#### 2.8.5 Theme 5: Dynamic Considerations Based on Experience and Reality

Older adults' accounts of contemplating healthcare‐seeking behaviors illustrate the interplay of accumulated knowledge, experiences, and social structures. This encompasses self‐awareness, experiences, and perceptions, social interactions, and access to resources.

Some participants expressed minimal interest in the disease itself but were primarily concerned with its symptoms or effects, such as pain. Certain participants suggested that prior self‐treatment practices may have had an impact. Furthermore, the factor mentioned most frequently by participants is the reluctance to disturb their children or worry about expenses, which also impacts their healthcare‐seeking behavior.

I don't want to bother my kids while they're working. My illness isn't that serious, and nothing really needs to be done. If I'm feeling unwell and they all rush back to take care of me, they won't be able to earn much money.

(P11)

I feel like going to the hospital is a waste of money if they can't fully cure me.

(P8)

When I had a toothache before, my daughter took me to the hospital, and the money just adds up so easily—it really makes me feel bad about spending it.

(P13)

Neighborhood support, family dynamics, and guidance from healthcare professionals were additional factors that impacted their healthcare‐seeking behaviors.

These methods, like using herbs, have been passed down through the generations. We learned them from our elders, and now that we're older, we'll pass them on to our children.

(P15)

My relatives saw that I wasn't feeling well, so they suggested I go to the hospital. At first, I didn't want to go, but they ended up forcing me.

(P8)

If the village doctor's treatment doesn't work for a long time, I suggest my relatives or neighbors see a fortune‐teller.

(P12)

Limited services and the availability of local health resources are factors that also impact the behavior of older individuals.

Back when we were young, transportation was difficult, and it was hard to get to the hospital. That's why many elderly people used herbs to treat our illnesses.

(P12)

It's great that we can find herbs on the nearby mountains—it saves us from having to go to the hospital.

(P14)

I don't speak Mandarin, and I don't even understand the local dialect in the county. I only speak our Dong language, so when I go to the hospital, they can't understand me, and I can't understand them.

(P12)

## 3 Discussion

This study is the first to investigate the healthcare‐seeking behaviors of ethnic minority older adults from the Dong nationality in China. The healthcare‐seeking behavior of older individuals in traditional minority villages is characterized by a dynamic process of “action‐interpretation‐evaluation.” The study identified five themes that reflect the healthcare‐seeking behavior of the elderly in traditional minority villages. Themes 1 and 2 focus on self‐care for minor diseases and seeking healthcare assistance for major illnesses, respectively. Themes 3 and 4 delve into the interpretation of health‐related factors and disease symptoms within the context of traditional ethnic culture. Theme 5 explores dynamic considerations informed by experience and reality. These themes contribute to a comprehensive understanding of healthcare‐seeking behaviors in older adults from traditional ethnic minority villages. Drawing on the symptom‐response theory and the study's key findings, a conceptual framework (refer to Figure 1) has been developed. This framework serves as a valuable reference for further exploration of healthcare‐seeking behavior among older individuals in traditional ethnic minority villages in China, as well as for the design of targeted interventions and relevant policies.

Our research highlights the practical implications of understanding and addressing healthcare‐seeking behavior among older individuals in traditional ethnic minority villages. The impact of knowledge‐experience perceptions, social interactions, and resources on healthcare‐seeking behavior aligns with the integrated symptom‐response framework, indicating that the process of action‐interpretation‐evaluation is influenced by these factors [13]. Ethnic minority populations typically exhibit low health literacy levels [18]. Previous studies have identified poverty, limited education, and residence in specific areas as barriers to healthcare‐seeking behaviors in older adults [19]. Our study reveals a generally low level of education among older adults in traditional ethnic minority villages, directly affecting their perceptions and concerns regarding health and medical knowledge, including a lack of emphasis on disease prevention.

The family serves as a cornerstone of social interaction for older individuals, with family support playing a significant role in influencing their healthcare‐seeking behavior as indicated in prior research [20]. A study by Ram et al. [21] highlighted the importance of positive role modeling and support from adult children in facilitating health behavior changes among older South Asian immigrants. In Chinese families, intergenerational relationships are shaped by Confucian ethics, emphasizing reciprocal transmission and filial piety [11]. The cultural values of family and filial piety form the foundation of China's traditional elderly care system [20]. Typically, older adults transfer resources to their children and depend on them in their later years, while adult children are expected to demonstrate filial piety by offering financial support and caregiving in their parents’ old age [11]. Research also suggests that ethnic minority older adults are inclined to seek health advice from village doctors, possibly due to the accessibility of these healthcare providers to rural residents [22]. Additionally, prior studies have shown that ethnic minorities are more likely to perceive their doctors as confidants or friends [23].

Our research indicates that ethnoreligious culture plays a dual role in shaping the healthcare‐seeking behaviors of ethnic minority older adults. The use of herbal medicines reflects the development of ethnomedicine, while spiritual factors, such as the importance of virtues and religious beliefs, can have a protective influence. The influence of Chinese philosophy, culture, and traditional Chinese medicine (TCM) significantly shapes the health concepts, practices, and disease attitudes of rural residents [24]. Additionally, healthcare‐seeking behaviors are significantly influenced by folk culture, with individuals evaluating their health based on past experiences, beliefs, physical status, and available social resources [24]. Often, individuals turn to traditional remedies like witchcraft and religious rituals initially, with modern medicine considered a last resort [25]. This reliance on traditional practices may lead to negative coping mechanisms among older adults when faced with illness, consequently increasing health risks.

Our study offers practical implications to enhance healthcare‐seeking behaviors among ethnic minority older adults at both individual and systemic levels. At the individual level, promoting a healthy lifestyle, leveraging the protective aspects of religious culture, enhancing health literacy through tailored education, and raising awareness about early disease detection and intervention are crucial. Systemically, reallocating medical resources, enhancing the primary care team's quality, empowering village doctors as health gatekeepers, and implementing healthcare policies for poverty alleviation are recommended to mitigate barriers to routine medical care, particularly in addressing challenges related to accessibility and affordability.

### 3.1 Study Limitations

The findings of this study on the older Dong Chinese population may not be extrapolated to other ethnic minority groups. Differences in dialects, local traditions, religious beliefs, and health practices among various regions could restrict the transferability of our findings to other minority communities in China [26]. Nevertheless, they offer insights into the healthcare‐seeking behaviors of ethnic minorities, thereby informing the development of transcultural healthcare practices [27]. It is important to note that the study exclusively focused on older adults as participants. Future research on this subject should involve gathering perspectives from family members, healthcare professionals, and policymakers to comprehensively assess the healthcare system and enhance our understanding of healthcare‐seeking behaviors among ethnic minorities.

## 4 Conclusion

This study elucidates the healthcare‐seeking behavior of older individuals in traditional ethnic minority villages, examining their action‐explanation‐evaluation process and its interplay with their knowledge‐experience paradigms, social interactions, and available resources [28]. The study findings advocate for the establishment of a comprehensive transcultural intervention system for ethnic minority elderly, emphasizing the enhancement of health knowledge and literacy, promotion of supportive social interactions, and optimization of medical and health resource distribution [29]. To further improve health outcomes in this population, enhancing village‐level healthcare through culturally competent training for village doctors and the provision of additional resources is essential [30]. Such measures may facilitate timely and effective interventions and help reduce reliance on self‐care following inadequate treatment experiences.

## Author Contributions

**Liutao Wu:** writing – original draft, data curation, investigation, visualization, formal analysis. **Runxi Xiao:** writing – original draft, data curation, writing – review and editing, visualization. **Yu Wu:** data curation, investigation, formal analysis. **Qingmei Huang:** methodology, conceptualization, supervision, project administration, writing – review and editing. **Yan Liang:** supervision, methodology, conceptualization, project administration, resources, funding acquisition, writing – review and editing.

## Ethics Statement

Both ethical approval and informed consent from all older adults were obtained for this study.

## Conflicts of Interest

The authors declare no conflicts of interest.

## Transparency Statement

The lead author Qingmei Huang, Yan Liang affirms that this article is an honest, accurate, and transparent account of the study being reported; that no important aspects of the study have been omitted; and that any discrepancies from the study as planned (and, if relevant, registered) have been explained.

## Data Availability

The data that support the findings of this study are available from the corresponding author upon reasonable request.
